# Four-dimensional Computed Tomography Imaging in Primary Hyperparathyroidism: Multireader Multicase Study of Both Neuroradiologists and General Radiologists of Imaging Approaches With Less Phases

**DOI:** 10.1097/RCT.0000000000001794

**Published:** 2025-08-28

**Authors:** Jorian P. Krol, Robin J.A. Duteweert, Laura N. Deden, Marie Louise E. Bernsen, Cornelis H. Slump, Wim J.G. Oyen

**Affiliations:** * Department of Radiology and Nuclear Medicine, Rijnstate Hospital, Arnhem; † Department of Robotics and Mechatronics, Faculty of Electrical Engineering, Mathematics and Computer Science, University of Twente, Enschede, The Netherlands; ‡ Department of Biomedical Sciences and Humanitas Clinical and Research Centre, Department of Nuclear Medicine, Humanitas University, Milan, Italy; § Department of Radiology and Nuclear Medicine, Radboud University Medical Centre, Nijmegen, The Netherlands

**Keywords:** primary hyperparathyroidism, four dimensional CT, multireader multicase study

## Abstract

**Objective:**

Primary hyperparathyroidism (PHPT) is commonly caused by parathyroid adenomas (PAs), and four-dimensional computed tomography (4DCT) is increasingly used for localising PAs due to its high sensitivity and specificity. However, the relative high radiation dose of 4DCT may limit its widespread use as first line imaging in some settings. A reduced phase protocol and enhancement maps, which highlight relative enhancement differences between the nonenhanced and arterial phases, have been proposed as ways to reduce radiation exposure without compromising diagnostic accuracy. This study aims to assess whether reduced 4DCT protocols can maintain diagnostic performance and if the enhancement map can further assist in adenoma localisation.

**Methods:**

This retrospective study included 27 PHPT patients, with both single and double adenomas, and some ectopic cases and 3 secondary HPT patients. Five-phase combinations derived from our institution’s 4-phase protocol were evaluated using a multireader, multicase approach involving experienced neuroradiologists and general radiologists. The phases tested included combinations of nonenhanced, arterial, venous, and delayed venous phases. An enhancement map was introduced as one of the phases. Readers were asked to identify adenomas and assign confidence levels. Performance metrics, including sensitivity, specificity, and area under the curve (AUC), were calculated, and noninferiority tests compared the results to the current 4-phase protocol.

**Results:**

Sensitivity of the total group was between 0.64 and 0.70 with a specificity between 0.94 and 0.97. AUC were between 0.80 and 0.84. All reduced phase combinations were noninferior to the 4-phase protocol. Neuroradiologists achieved noninferior results with 1-phase to 3-phase protocols, while general radiologists required at least 3-phases. The enhancement map did not improve sensitivity or specificity, although readers found it useful as a supplementary tool for identifying lesions. Artefacts, especially in ectopic locations, reduced its effectiveness.

**Conclusions:**

This study supports the use of reduced 4DCT protocols for PHPT. A 1-phase or 2-phase protocol is recommended for experienced radiologists, while a 3-phase protocol is suitable for less experienced radiologists.

## Graphical abstract

**Figure:**
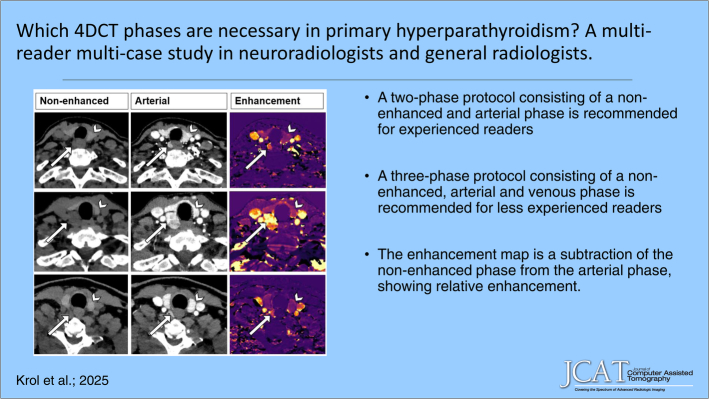

Primary hyperparathyroidism (PHPT) is an endocrine disorder that affects between 1 and 10 cases per thousand adults, with prevalence to 3.4% in postmenopausal women.^1,2^ PHPT is caused by excessive parathyroid hormone (PTH) secretion by single or multiple hyperfunctional parathyroid glands.^3,4^ Most individuals have 4 parathyroid glands, which are typically located along the thyroid lobes.^5^ Ectopic located glands, which can be found anywhere from the mandible angle to the mediastinum, is observed in 16% of patients with PHPT due to abnormal embryonal migration.^6,7^ In majority of cases (80%–85%), PHPT is caused by a solitary parathyroid adenoma (PA). In remaining cases (15%–20%), multiglandular disease is the underlying cause.^8,9^

Currently, four-dimensional computed tomography (4DCT) is the preferred first-line imaging modality for localizing PAs in an increasing number of hospitals.^10–12^ Primary rationale for increased 4DCT use is superior sensitivity and specificity, faster acquisition and increased reproducibility as compared with ultrasound (US) and ^99m^Tc-Sestamibi SPECT (Tc-MIBI).^13^ Nevertheless, a disadvantage of 4DCT is its relatively higher radiation dose as compared with US or MIBI.^14,15^

In 2006, Rodgers et al^16^ initially delineated a multiphase 4DCT protocol. Mortenson et al^17^ first described a 4-phase 4DCT protocol in 2008. This protocol comprised of nonenhanced, arterial, venous and delayed venous phases. This protocol is currently used in our institution. However, there has been a considerable discrepancy in number of phases and contrast phase timing described in literature, as evidenced by the systematic review conducted by Kluijfhout et al18 and Raeymaeckers et al.^19^ A survey by Hoang et al^20^ showed that a 3-phase protocol including a nonenhanced phase, is most commonly used. Several studies have proposed a reduced 4DCT as a means to reduce radiation dose to the patient.^21–27^ However, most studies have only compared 2 distinct protocols. Moreover, majority of studies used readers with extensive experience in 4DCT, namely neuroradiologists, who may require fewer phases to achieve the same sensitivity and specificity as less experienced radiologists.

An additional method for reducing radiation dose is enhancement map utilization as a contrast phase replacement. This constitutes a variation of the subtraction map previously described.^28^ The enhancement map demonstrates relative enhancement of each voxel between nonenhanced and arterial phase. In theory, this demonstrates a relative enhancement increase of the PA compared with the thyroid gland, which could also enhance PA detection, particularly when enhancement observed in the contrast phases is comparable to that of the thyroid gland. Vance-Daniel et al^29^ showed in their study that there is a significant increase in enhancement of PA compared with thyroid tissue, stating that percentage difference in HU may be more useful to identify PAs. This additional information can be derived from phases that are already used, thus reducing the necessity for venous and delayed venous phases, which could potentially result in a reduction in radiation dose. This study presents initial findings from the clinical utilization of the enhancement map.

This study uses a multireader multicase (MRMC) methodology to examine efficacy of 5 distinct combinations of 4DCT phases across a diverse cohort of readers, encompassing varying expertise levels in 4DCT. This study aims to ascertain whether the number of phases can be reduced and, if so, whether optimal number of reduced phases varies according to the reader’s level of experience.

## METHODS

### Patient and Data Acquisition

Four-dimensional computed tomography scans from patients with biochemically and histopathologically proven PHPT were retrospectively included from May 2020 until March 2023. From this larger data set, the required number of eutopic single adenomas, double adenomas and ectopic adenomas was first determined, according to existing literature.^30,31^ Subsequently, the cases were sequentially selected from this larger data set, ensuring each type of adenoma was represented according to our predefined criteria. A small number of cases with confirmed secondary hyperparathyroidism were included as 4DCT without candidate parathyroid adenoma. The study was approved by the institutional research ethics committee (Research number 2023-2321).

### Scan Protocol

**FIGURE 1:**
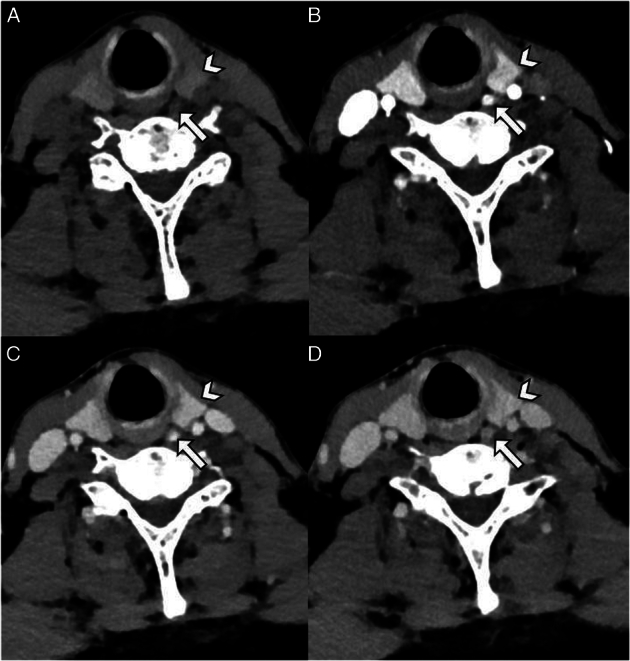
A–D, Example of the current 4DCT protocol (nonenhanced (A), arterial (B), venous (C) and delayed venous (D) phase). Arrowhead points to the thyroid gland, arrow points toward the parathyroid adenoma.

All patients were scanned on the IQon dual-energy CT scanner (Philips Healthcare) with 0.9 mm slice thickness, 120 kVp tube voltage and a tube current-exposure time product value between 27mAs and 88mAs. Four-dimensional computed tomography protocol at our institution comprises of a nonenhanced phase, followed by administrating 90 to 120 mL iodine-based intravenous contrast agent (Xenetix300, Guerbet, Villepinte, France) at 3.5-5.0 mL/s flow rate depending on weight. The proximal part of the descending aorta is used for bolus tracking with a 150 HU threshold. Arterial, venous and delayed venous phases are acquired at 10, 40 and 85 seconds postthreshold delay, respectively. Patients were placed in a supine position with extension of the neck and lowering of the shoulders. The average dose length product of this protocol was 497 mGy*cm, with a calculated effective dose of 5.6 mSv. A 4DCT-protocol example is provided in Figure 1.

### Multireader Multicase Structure

**FIGURE 2:**
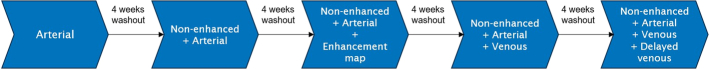
Phase combination in each of the 5 rounds starting with the arterial phase. Washout period of 4 weeks in between phases.

The study comprised of 5 different rounds, each round consisting of a different phase combinations. First a power analysis was conducted to ascertain the required number of readers and number of scans to be reviewed per round using iMRMC software.^32^ An effect size of 0.05 for a factorial, noninferiority study with both random readers and cases showed a power of 0.8 for at least 9 readers with 30 cases per round using variance components, covariances and correlation numbers obtained from a previously performed small pilot study.^33^ All readers (7 neuroradiologists and 6 general radiologists) were provided with an instructional presentation that covered the theoretical basis of 4DCT, presented epidemiologic data (such as prevalence of single adenomas, double adenomas and ectopic locations) from literature, and included several illustrative examples of PAs. Subsequently, readers were provided with a teaching file comprising 19 validated cases of PHPT, which they could utilize for practice in reviewing such scans. This was done to minimize learning curve impact, particularly in the group with less experience in 4DCT. Before the third round, another presentation was available, providing theoretical information about the enhancement map and a teaching file containing enhancement map examples of previous PHPT cases, which they were required to review. After this, readers proceeded to the MRMC study. The study comprised of 5 different rounds. In accordance with recommendations described by Obuchowsky and Bullen,^34^ the sequence of the rounds and phase combination were based on expected benefit from literature (ie, the arterial phase was hypothesized to be the most beneficial in concordance with literature, followed by the noncontrast phase, etc). As shown in Figure 2, the first round consisted solely of the arterial phase, while in subsequent rounds additional phases were incorporated. To mitigate the potential for recall bias, additional information was incorporated in each successive round. A minimum 4-week washout period was observed. Same anonymized cases were reviewed each round, in a random order. Four-dimensional computed tomography scans were reviewed by the readers using the Sectra PACS environment (Sectra AB), which they were very familiar with. Layouts were preset, consisting of axial images on the left screen and coronal images on the right screen. Readers filled in a questionnaire during the review process, inquiring about presence of one or more PAs and location of each lesion. Lesions were classified into 4 quadrants relative to the thyroid gland or an ectopic location. Readers were required to assign a confidence level to each lesion, categorized into 4 levels (0%–25%, 25%–50%, 50%–75%, and 75%–100%). Furthermore, readers were requested to indicate lesions in the images with a marker, thus enabling principal investigators (J.P.K. and R.J.A.D.) to verify the location indicated in the questionnaire. PA location was determined by reference the surgical report and histopathologic confirmation, and this constituted as the ground truth for each case.

### Enhancement Map

**FIGURE 3:**
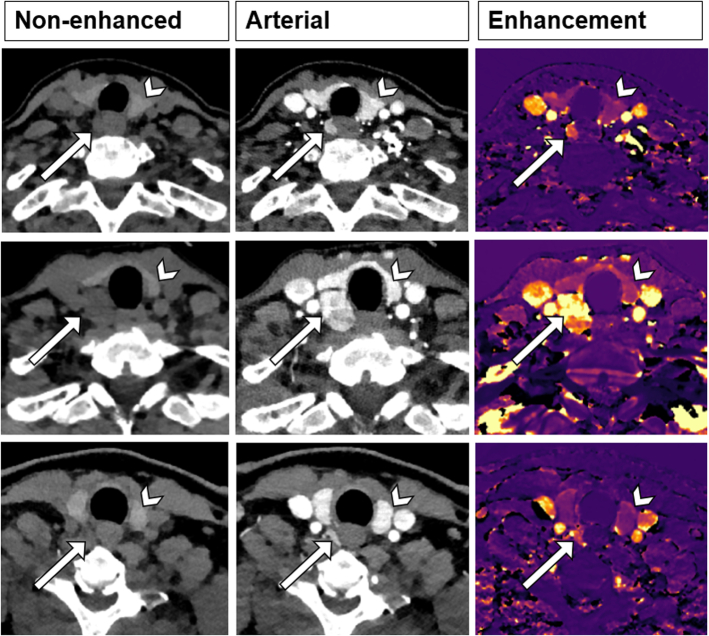
Example of 3 different cases with a nonenhanced phase, arterial phase and enhancement map. Arrowhead shows the thyroid gland, arrow shows the parathyroid adenoma.

In round 3, a novel reconstruction technique was introduced, utilizing only data obtained from nonenhanced and arterial phases, being a variation of the subtraction map previously described.^28^ The enhancement map demonstrated relative enhancement per voxel between nonenhanced and arterial phases. It should be noted that all readers had limited experience with the enhancement map. A comprehensive presentation regarding underlying theory and 5 illustrative cases was provided to the readers, and they had access to a teaching file containing 15 cases. It was required for the readers to complete the teaching file before commencing round 3. See Figure 3 for 3 typical enhancement map examples.

### Data Analysis and Statistics

Data were analyzed on a localization basis, with each case divided into 5 locations/quadrants (upper left, upper right, lower left, lower right, and ectopic) to facilitate a per-lesion analysis. A true positive result was defined as correct quadrant identification by the reader for each lesion. A true negative was defined as the correct quadrant exclusion when no lesion was present in that quadrant. A false negative was defined as failure to mark a quadrant despite presence of a lesion in that quadrant. A false positive was defined as marking of a quadrant in absence of a lesion. In accordance with recommendations of Obuchowsky and Bullen, individual sensitivity and specificity with 95% CI, grouped sensitivity and specificity with 95% CI, AUCs per reader per round, AUCs per group per round and total AUCs per round were calculated. Noninferiority test was conducted to evaluate performance of each round in comparison to round 5 (current protocol in our institution) for both grouped and total AUCs. In instances where a round was deemed to be noninferior to round 5, a superiority test was also conducted to ascertain whether the round in question was superior to round 5. *P*-value of 0.05 was considered statistically significant, and noninferiority margin was also set at 0.05. Statistical analyses were conducted using R software (R version 4.0.3; R Foundation for Statistical Computing), with iMRMC package utilized for MRMC statistical analyses, including noninferiority test.^32^

## RESULTS

### Patient and Data Acquisition

Thirty patients were selected for inclusion in the data set. Twenty-seven patients had PHPT, caused by surgically and histopathologically proven PA (90%). Three cases (10%) were proven secondary hyperparathyroidism with a 4DCT without candidate parathyroid adenoma. Of the 27 cases with PHPT, 24 cases (89%) had a single adenoma, and 3 cases (11%) had a double adenoma. Of the 24 cases with a single adenoma, 3 cases were in an ectopic location (12.5%). Patient characteristics are shown in Supplemental Table 1 (Supplemental Digital Content 1, http://links.lww.com/RCT/A368).

### Performance Metrics and Noninferiority Analysis

**FIGURE 4:**
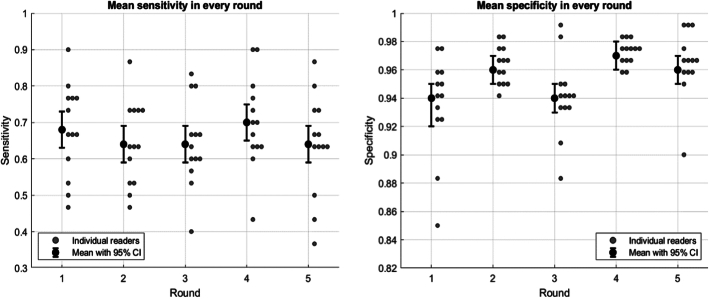
Individual sensitivity and specificity per round with mean and 95% CI, respectively.

Sensitivity and specificity for each reader are illustrated in Figure 4, and individual sensitivity and specificity with 95% CI are included in Supplemental Table 2 (Supplemental Digital Content 2, http://links.lww.com/RCT/A369). Table 1 presents individual AUC values for each round, along with total mean AUC and 95% CI for each round. Table 2 presents grouped sensitivity, specificity, and AUC with 95% CI per round. Mean sensitivity for the whole group was between 64% and 70%, with specificity between 94% and 97%. Highest AUC was observed in round 4, while lowest was seen in round 3 with AUCs between 0.80 and 0.84. Noninferiority test demonstrated significant noninferiority of all rounds in comparison with round 5, the current protocol (*P* < 0.001–0.011).

**TABLE 1:** Individual AUC Per Round

Reader	Group*	Round 1	Round 2	Round 3	Round 4	Round 5
Reader 01	N	0.87	0.85	0.81	0.79	0.81
Reader 02	N	0.78	0.75	0.77	0.80	0.74
Reader 03	N	0.88	0.85	0.90	0.95	0.86
Reader 04	G	0.78	0.75	0.78	0.82	0.81
Reader 05	G	0.78	0.72	0.78	0.71	0.68
Reader 06	G	0.71	0.80	0.66	0.85	0.67
Reader 07	G	0.83	0.80	0.78	0.80	0.80
Reader 08	N	0.90	0.81	0.82	0.81	0.83
Reader 09	G	0.85	0.86	0.87	0.89	0.85
Reader 10	N	0.76	0.79	0.81	0.84	0.89
Reader 11	N	0.86	0.85	0.75	0.87	0.80
Reader 12	N	0.94	0.92	0.90	0.94	0.93
Reader 13	G	0.73	0.73	0.78	0.84	0.82
	Mean	0.82 (0.76, 0.88)	0.81 (0.74, 0.87)	0.80 (0.73, 0.86)	0.84 (0.78, 0.90)	0.81 (0.75, 0.87)

Readers are divided in 2 groups: neuroradiologists (N) and general radiologists (G). Mean AUC per round with 95% CI is included.

* N indicates neuroradiologist; G, general radiologist.

**TABLE 2:** Mean Sensitivity, Specificity, AUC Per Round With 95%CI

	Round 1	Round 2	Round 3	Round 4	Round 5
All readers					
Sensitivity	0.68 (0.63, 0.73)	0.64 (0.59, 0.69)	0.64 (0.59, 0.69)	0.70 (0.65, 0.75)	0.64 (0.59, 0.69)
Specificity	0.94 (0.92, 0.95)	0.96 (0.95, 0.97)	0.94 (0.93, 0.95)	0.97 (0.96, 0.98)	0.96 (0.95, 0.97)
AUC	0.82 (0.76, 0.88)	0.81 (0.74, 0.87)	0.80 (0.73, 0.86)	0.84 (0.78, 0.90)	0.81 (0.75, 0.87)
ΔAUC with round 5	0.013 (−0.028, 0.054)	0.001 (0.036, 0.037)	−0.007 (−0.037, 0.024)	0.033 (−0.003, 0.069)	—
*P*	0.006	0.011	0.010	<0.001	—
Outcome	Noninferior	Noninferior	Noninferior	Noninferior	—
Neuroradiologists					
Sensitivity	0.73 (0.67, 0.79)	0.69 (0.62, 0.75)	0.68 (0.61, 0.74)	0.73 (0.67, 0.79)	0.69 (0.62, 0.75)
Specificity	0.95 (0.93, 0.96)	0.96 (0.95, 0.97)	0.95 (0.93, 0.96)	0.97 (0.96, 0.98)	0.97 (0.96, 0.98)
AUC	0.85 (0.77, 0.92)	0.83 (0.76, 0.90)	0.82 (0.75, 0.89)	0.86 (0.78, 0.93)	0.84 (0.77, 0.91)
ΔAUC with round 5	0.016 (−0.048, 0.081)	−0.005 (−0.046, 0.037)	−0.015 (−0.057, 0.027)	0.019 (−0.032, 0.070)	—
*P*	0.046	0.038	0.086	0.014	—
Outcome	Noninferior	Noninferior	Possibly inferior	Noninferior	—
General radiologists					
Sensitivity	0.62 (0.54, 0.69)	0.58 (0.51, 0.66)	0.60 (0.52, 0.67)	0.66 (0.59, 0.73)	0.58 (0.50, 0.65)
Specificity	0.92 (0.90, 0.94)	0.97 (0.95, 0.98)	0.93 (0.91, 0.95)	0.97 (0.96, 0.98)	0.96 (0.94, 0.97)
AUC	0.78 (0.71, 0.85)	0.78 (0.70, 0.85)	0.77 (0.70, 0.85)	0.82 (0.75, 0.89)	0.77 (0.70, 0.84)
ΔAUC with round 5	0.009 (−0.059, 0.077)	0.007 (−0.072, 0.087)	0.003 (−0.053, 0.060)	0.048 (−0.024, 0.121)	—
*P*	0.076	0.128	0.061	0.017	—
Outcome	Possibly inferior	Possibly inferior	Possibly inferior	Noninferior	—

Data are also split per group. Difference in AUC with round 5 including 95% CI is shown per round. Noninferiority test *P*-value is also included, *P*-value <0.05 is significant.

### Neuroradiologists Versus General Radiologists

**FIGURE 5:**
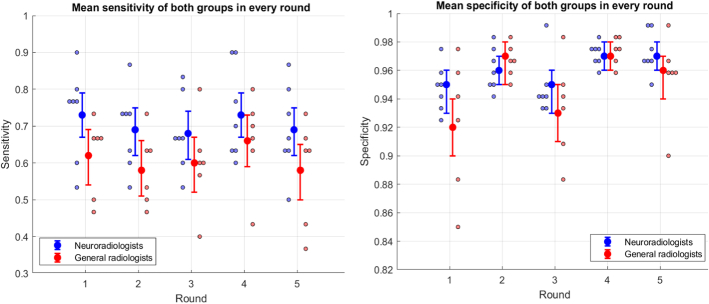
Individual sensitivity and specificity per round with mean and 95% CI per group, respectively. Neuroradiologists are shown in blue, general radiologists are shown in red.

Readers were divided into 2 groups based on their experience with 4DCT, resulting in a neuroradiologist and general radiologist group. Sensitivity and specificity for both groups including the mean with 95% CI are illustrated in Figure 5. Highest sensitivity was observed for neuroradiologists in rounds 1 and 4. For general radiologists, highest sensitivity was observed in round 4. Highest specificity was observed for both groups in rounds 2, 4, and 5. Mean AUC with 95% CI was plotted for each group. Noninferiority test in the neuroradiologist group showed a significant noninferiority of rounds 1, 2 and 4 compared with round 5 (*P* = 0.014–0.046). Noninferiority test in the general radiologist group showed significant noninferiority of round 4 compared with round 5 (*P* = 0.017). No rounds were identified as being superior to round 5 in either group (*P* = 0.075–0.408). Further details are provided in Table 2. None of the general readers showed increasing sensitivity or specificity in the consecutive rounds, therefore, no apparent learning curve was detected.

### Enhancement Map

All readers completed the questionnaire. Questionnaire results are presented in Supplemental Figure 1 (Supplemental Digital Content 3, http://links.lww.com/RCT/A370), with mean responses indicated by the red dots and range of the responses shown by a red line. Questionnaire results indicated arterial phase was most frequently used as the first phase for detection, followed by the enhancement map, while nonenhanced phase was least used. The second question revealed readers expressed greater confidence in locating the PA by using the enhancement map. Some radiologists indicated they used the enhancement map as a screening tool to further evaluate potential lesions in the arterial and nonenhanced phases. Readers identified artifact reduction and absence of a color scale as areas of improvement. However, readers did indicate they would like to use the enhancement map in daily practice after optimization.

## DISCUSSION

In this multireader multicase study, potential 4DCT phase reduction in preoperative PA localization in PHPT patients was analyzed. Overall, all rounds were significantly noninferior to the current 4-phase protocol. In the more experienced reader group, 1-phase, 2-phase, and 3-phase protocols were significantly noninferior compared with the current 4-phase protocol. In the less experienced reader group, only the 3-phase protocol was significantly noninferior compared with the current 4-phase protocol. No phase combination was significantly superior to the current 4-phase protocol. The enhancement map did not increase sensitivity or specificity. Nevertheless, readers considered the enhancement map to be of value.

Results of our study indicate that not all 4 phases in the 4DCT protocol are truly necessary. Following this study, the 4DCT protocol in our institution has been adjusted to a 2-phase protocol. This 4-phase protocol was previously described by Mortenson et al^17^ and was used to increase differentiation between thyroid, lymph node and parathyroid glands. This finding is consistent with previous studies in literature. A number of studies have recommended 1-phase, 2-phase, or 3-phase protocols. In their systematic review and meta-analysis, Kluijfhout et al^18^ concluded that a 3-phase protocol was optimal for sensitivity. A 2-phase protocol, comprising of a nonenhanced and arterial phase, was proposed by Campbell and colleagues, Ramirez and colleagues, and Noureldine and colleagues. This approach yielded comparable outcomes to those observed in their 4-phase protocol.^24–26^ Leiva-Salinas et al^27^ demonstrated that a 2-phase protocol from a monophasic dual-energy CT acquisition, utilizing a virtual noncontrast phase in combination with an arterial phase, yielded comparable results to their biphasic protocol. In correspondence to noninferiority of the arterial phase only for neuroradiologists in our results, some previous studies have proposed an arterial 1-phase protocol. Moron et al^35^ showed a localization sensitivity of 81.4% in 32 PHPT patients. Raghavan et al^21^ demonstrated that diagnostic accuracy was adequate when using arterial images alone (90.5% lateralization and 91.5% localization accuracy). Silver et al^36^ demonstrated that a 1-phase protocol showed comparable sensitivity to a 4-phase protocol. In contrast, Bahl et al^37^ stated that the absence of a nonenhanced phase could result in the misdiagnosis of 22% of PAs that demonstrate similar enhancement to the thyroid gland on both the arterial and venous phase. Previous studies by Galvin et al^38^ and Sho et al^39^ have analyzed and described the factors associated with missed lesions. Most missed lesions would not necessarily be detected on a (delayed) venous phase, except for the potential streak artifact from the contrast bolus in the case of an inferior position of the PA. Therefore, based on our findings and results from literature, we propose a 2-phase protocol for experienced neuroradiologists consisting of a nonenhanced and arterial phase.

In numerous studies, data sets were interpreted by a limited number of neuroradiologists with considerable 4DCT expertise.^40,41^ As illustrated by our data, radiologists with high level of experience do require fewer phases to achieve noninferior accuracy compared with radiologists with a lower 4DCT experience level. Randall and colleagues also describes higher detection rates when radiologists are already familiar with the typical PA appearance and location. Readers showed a learning curve in identifying adenomas, particularly when ectopic.^42^ Sepahdari et al^43^ described an initial experience in starting with a 3-phase 4DCT protocol in 2 inexperienced readers, with a prospective accuracy of 78%. To the best of our knowledge, there are no other MRMC studies comparing performance of more and less experienced radiologists in PA detection using 4DCT or studies comparing different 4DCT protocols in less experienced radiologists. Furthermore, previous literature could not provide a sufficient protocol proposal for less experienced radiologists or institutions that would want to introduce a 4DCT protocol. Therefore, based on our findings, we propose a 3-phase protocol for radiologists with less experience in 4DCT or when an institution would introduce a 4DCT protocol in their PHPT imaging pathway, consisting of a nonenhanced, arterial and venous phase.

The enhancement map as was used in round 3, combined with the nonenhanced and arterial phase was hypothesized to help detection and increase tissue differentiation by combining information from the nonenhanced and arterial phase. In contrast, Round 3 showed lowest sensitivity and specificity in both groups. However, readers were positive about the enhancement map. Some readers used the enhancement map as screening tool, to guide where a PA might be located, others used it to determine whether the lesion showed avid enhancement compared with the thyroid gland. However, it was observed that the enhancement map is susceptible to artifacts as occur in the noncontrast or arterial phase. Especially PAs caudally from the thyroid were susceptible to beaming artifacts and showed a lower relative enhancement compared with the thyroid. Some readers did not recognize the artifacts and marked this a false negative. More experience with enhancement maps is necessary to increase detection rate using the enhancement map. Moreover, additional research in how to reduce artifacts is necessary, both in conventional 4DCT phases as in the enhancement map.

The present study is not without limitations. The study was conducted at a single institution with a relative small subset, which may limit extrapolation of our findings including the noninferiority results to other settings. However, use of readers with varying experience levels did introduce a greater degree of variation to increase generalizability. Moreover, the number of phase combinations that could be investigated was limited, necessitating a subset selection based on literature review findings and researchers’ prior experience at this institution. As a result, the arterial phase was included in all image sets, therefore, its necessity is assumed from previous literature, but not assessed in this study. The sensitivity and specificity of round 5 were lower for both groups compared with round 4, even though no superiority was shown. Our hypothesis is that the addition of a delayed venous phase could confuse radiologists, especially less experienced radiologists. In the delayed venous phase, the adenoma did not always show further wash-out, or it could look more similar to a normal lymph node, increasing confusion and lowering sensitivity. The relative small number of cases each round cannot fully represent the range of challenging PHPT localizations, however, the percentage of challenging cases in this data set has been selected to represent real-world practice. Given that half of the readers had limited to no 4DCT experience, it was anticipated a learning curve would be evident in the successive rounds. To minimize learning curve impact, all readers were provided with an informative presentation and a teaching file containing examples. It was hypothesized that the learning curve would exert less influence than recall bias. Therefore, phase order was not randomized and only in each successive round, additional information was presented.

## CONCLUSION

Four-dimensional CT is becoming increasingly prevalent as a preoperative imaging modality for PHPT, with a multitude of protocols documented in literature. Our multireader multicase study evaluated efficacy of different phase combinations in both experienced and less experienced readers. In institutions with radiologists with extensive 4DCT experience, a 2-phase protocol is recommended, comprising of a nonenhanced and arterial phase. Conversely, in institutions with radiologists who are less experienced in or new to 4DCT, a 3-phase protocol, comprising of a nonenhanced, arterial and venous phase, is recommended.

## Supplementary Material
