# Supplementary material for: Four-dimensional Computed Tomography Imaging in Primary Hyperparathyroidism: Multireader Multicase Study of Both Neuroradiologists and General Radiologists of Imaging Approaches With Less Phases
Source: J Comput Assist Tomogr. 2025 Aug 28;50(2):331–8. doi: 10.1097/RCT.0000000000001794 (PMC12986040; doi:10.1097/RCT.0000000000001794)
Supplement: Supplementary file 1 [file rct-50-331-s001.docx]

#### Supplementary Table 1: Patient characteristics in the dataset.

| Patient characteristics (n = 30) | |
| --- | --- |
| Age, mean (SD) | 61 (11) |
| Female, n (%) | 25 (83.3) |
| Weight (kg), mean (SD) | 72.5 (17.5) |
| Size of adenoma (mm), mean (SD) | 12.6 (8.8) |
| Number of adenomas |  |
| Single adenoma, n (%) | 24 (80) |
| Double adenoma, n (%) | 3 (10) |
| No adenoma, n (%) | 3 (10) |
| Location of adenomas |  |
| Upper left, n (%) | 3 (11.1) |
| Bottom left, n (%) | 10 (37.0) |
| Upper right, n (%) | 1 (3.7) |
| Bottom right, n (%) | 7 (25.9) |
| Ectopic, n (%) | 3 (11.1) |
| Bottom left & bottom right, n (%) | 2 (7.4) |
| Upper left & bottom right, n (%) | 1 (3.7) |
| PTH blood level (pmol/L) |  |
| PTH pre-operative, mean (SD) | 25.5 (19.7) |
| PTH post-operative, mean (SD) | 5.0 (5.3) |
