# Supplementary material for: Four-dimensional Computed Tomography Imaging in Primary Hyperparathyroidism: Multireader Multicase Study of Both Neuroradiologists and General Radiologists of Imaging Approaches With Less Phases
Source: J Comput Assist Tomogr. 2025 Aug 28;50(2):331–8. doi: 10.1097/RCT.0000000000001794 (PMC12986040; doi:10.1097/RCT.0000000000001794)
Supplement: Supplementary file 2 [file rct-50-331-s002.docx]

#### Supplementary Table 2: Individual sensitivity and specificity with 95% CI per round. Total mean with 95% CI included. * N = neuroradiologist. G = general radiologist.

| **Specificity** | Round 5 | 0.99  (0.95, 1.00) | 0.97  (0.92, 0.99) | 0.97  (0.92, 0.99) | 0.97  (0.92, 0.99) | 0.99  (0.95, 1.00) | 0.90  (0.83, 0.95) | 0.97  (0.92, 0.99) | 0.99  (0.95, 1.00) | 0.96  (0.91, 0.99) | 0.97  (0.92, 0.99) | 0.94  (0.88, 0.98) | 0.97  (0.92, 0.99) | 0.96  (0.91, 0.99) | **0.96**  (0.95, 0.97) |
| --- | --- | --- | --- | --- | --- | --- | --- | --- | --- | --- | --- | --- | --- | --- | --- |
|  | Round 4 | 0.97  (0.93, 0.99) | 0.97  (0.92, 0.99) | 0.98  (0.94, 1.00) | 0.97  (0.93, 0.99) | 0.97  (0.93, 0.99) | 0.96  (0.91, 0.99) | 0.97  (0.92, 0.99) | 0.97  (0.93, 0.99) | 0.98  (0.94, 1.00) | 0.97  (0.93, 0.99) | 0.96  (0.91, 0.99) | 0.96  (0.91, 0.99) | 0.98  (0.94, 1.00) | **0.97**  (0.96, 0.98) |
|  | Round 3 | 0.94  (0.88, 0.98) | 0.93  (0.87, 0.97) | 0.99  (0.95, 1.00) | 0.93  (0.87, 0.97) | 0.95  (0.89, 0.98) | 0.88  (0.81, 0.93) | 0.94  (0.88, 0.98) | 0.95  (0.89, 0.98) | 0.91  (0.84, 0.95) | 0.94  (0.88, 0.98) | 0.94  (0.88, 0.98) | 0.93  (0.87, 0.97) | 0.98  (0.94, 1.00) | **0.94**  (0.93, 0.95) |
|  | Round 2 | 0.95  (0.89, 0.98) | 0.96  (0.91, 0.99) | 0.95  (0.89, 0.98) | 0.97  (0.92, 0.99) | 0.97  (0.92, 0.99) | 0.95  (0.89, 0.98) | 0.97  (0.93, 0.99) | 0.97  (0.93, 0.99) | 0.98  (0.94, 1.00) | 0.98  (0.94, 1.00) | 0.94  (0.88, 0.98) | 0.97  (0.92, 0.99) | 0.96  (0.91, 0.99) | **0.96**  (0.95, 0.97) |
|  | Round 1 | 0.96  (0.91, 0.99) | 0.93  (0.87, 0.97) | 0.95  (0.89, 0.98) | 0.88  (0.81, 0.93) | 0.85  (0.77, 0.91) | 0.92  (0.86, 0.97) | 0.97  (0.93, 0.99) | 0.97  (0.93, 0.99) | 0.94  (0.88, 0.98) | 0.95  (0.89, 0.98) | 0.94  (0.88, 0.98) | 0.92  (0.86, 0.97) | 0.96  (0.91, 0.99) | **0.94**  (0.92, 0.95) |
| **Sensitivity** | Round 5 | 0.63  (0.44, 0.80) | 0.50  (0.31, 0.69) | 0.73  (0.54, 0.88) | 0.63  (0.44, 0.80) | 0.37  (0.20, 0.56) | 0.43  (0.25, 0.63) | 0.63  (0.44, 0.80) | 0.67  (0.47, 0.83) | 0.73  (0.54, 0.88) | 0.80  (0.61, 0.92) | 0.63  (0.44, 0.80) | 0.87  (0.69, 0.96) | 0.67  (0.47, 0.83) | **0.64**  (0.59, 0.69) |
|  | Round 4 | 0.60  (0.41, 0.77) | 0.63  (0.44, 0.80) | 0.90  (0.73, 0.98) | 0.67  (0.47, 0.83) | 0.43  (0.25, 0.63) | 0.73  (0.54, 0.88) | 0.63  (0.44, 0.80) | 0.63  (0.44, 0.80) | 0.80  (0.61, 0.92) | 0.70  (0.51, 0.85) | 0.77  (0.58, 0.90) | 0.90  (0.73, 0.98) | 0.70  (0.51, 0.85) | **0.70**  (0.65, 0.75) |
|  | Round 3 | 0.67  (0.47, 0.83) | 0.60  (0.41, 0.77) | 0.80  (0.61, 0.92) | 0.63  (0.44, 0.80) | 0.60  (0.41, 0.77) | 0.40  (0.23, 0.59) | 0.60  (0.41, 0.77) | 0.67  (0.47, 0.83) | 0.80  (0.61, 0.92) | 0.67  (0.47, 0.83) | 0.53  (0.34, 0.72) | 0.83  (0.65, 0.94) | 0.57  (0.37, 0.75) | **0.64**  (0.59, 0.69) |
|  | Round 2 | 0.73  (0.54, 0.88) | 0.53  (0.34, 0.72) | 0.73  (0.54, 0.88) | 0.53  (0.34, 0.72) | 0.47  (0.28, 0.66) | 0.63  (0.44, 0.80) | 0.63  (0.44, 0.80) | 0.63  (0.44, 0.80) | 0.73  (0.54, 0.88) | 0.60  (0.41, 0.77) | 0.73  (0.54, 0.88) | 0.87  (0.69, 0.96) | 0.50  (0.31, 0.69) | **0.64**  (0.59, 0.69) |
|  | Round 1 | 0.77  (0.58, 0.90) | 0.60  (0.41, 0.77) | 0.77  (0.58, 0.90) | 0.67  (0.47, 0.83) | 0.67  (0.47, 0.83) | 0.47  (0.28, 0.66) | 0.67  (0.47, 0.83) | 0.80  (0.61, 0.92) | 0.73  (0.54, 0.88) | 0.53  (0.34, 0.72) | 0.77  (0.58, 0.90) | 0.90  (0.73, 0.98) | 0.50  (0.31, 0.69) | **0.68**  (0.63, 0.73) |
| * | | N | N | N | G | G | G | G | N | G | N | N | N | G |  |
|  |  | Reader 01 | Reader 02 | Reader 03 | Reader 04 | Reader 05 | Reader 06 | Reader 07 | Reader 08 | Reader 09 | Reader 10 | Reader 11 | Reader 12 | Reader 13 | Mean |
