# Supplementary material for: Four-dimensional Computed Tomography Imaging in Primary Hyperparathyroidism: Multireader Multicase Study of Both Neuroradiologists and General Radiologists of Imaging Approaches With Less Phases
Source: J Comput Assist Tomogr. 2025 Aug 28;50(2):331–8. doi: 10.1097/RCT.0000000000001794 (PMC12986040; doi:10.1097/RCT.0000000000001794)
Supplement: Supplementary file 3 [file rct-50-331-s003.docx]

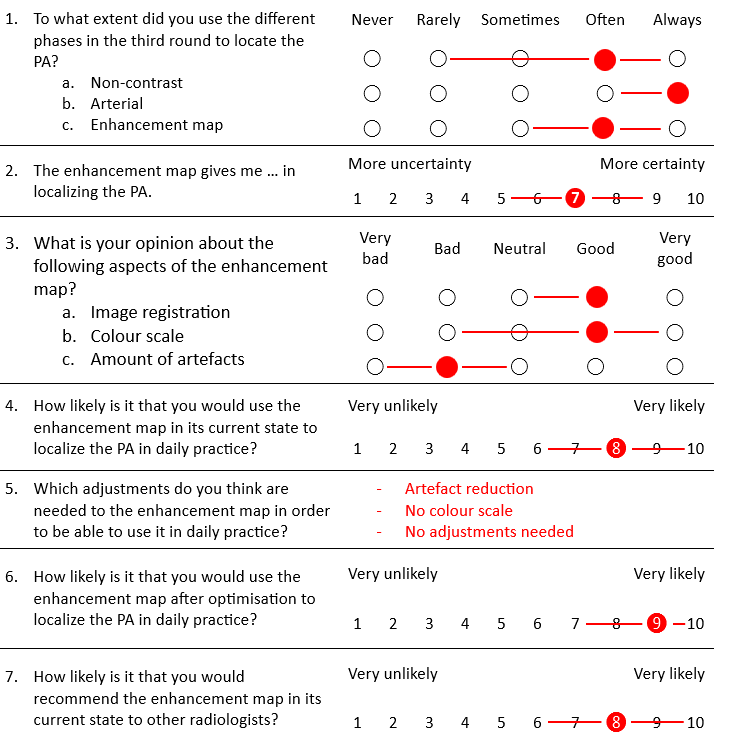


Supplementary Figure 1: The questionnaire including answers of all readers. Mean answer is shown as a red dot with the line showing the spread of answers.
